# The Professional Troubles of the Times

**Published:** 1872-01

**Authors:** 


### THE PROFESSIONAL TROUBLES OF THE TIMES.

Professional troubles are rife in the land—from Massachusetts to California, and from New York to Georgia. Even the capital of the country, sends its medical troubles over the telegraphic wires to repeat the unfortunate story.

In all these mutterings of opposing forces, there must be a latent cause, let loose here and there like Satan unchained, to reveal the thinness of the crust that overlies the crater ready to engulph the hopes, honor and interests of the profession, in ruin. These outcroppings send forth no uncertain indications of the deep moral and professional decay sapping, alike, the foundations of society and science. And yet, unopposed and unresisted, they would have remained hid from the keenest eye until the accumulating forces of error and corruption had swept away, with its besom of destruction, the last vestige of principle, honor and truth. Of all heroes, the possessor of a God-like soul, nerved by an immovable and unflinching integrity, under the holy impulse of an unconquered and unconquerable spirit, is the grandest, sublimest of earth. The shock of a battle, more terrific than any encounters of physical foes, may vibrate in Satanic fury above and around him, when the hosts of Apollyon shall pour forth from pandemonium armed with hellish vengence for his overthrow and ruin. There are moral heroes, over whose triumphs no peans have been sung—heroes as grand in their unyielding resistance to error as the mightiest conquerors enrolled on history's page. Among these we are proud to number the valient and true of the medical profession.

Great emergencies reveal great men, and times of trouble evoke the energies and marshal the resources of the truly good and brave, while the craven falter and fall before the march of unresisted wrongs. Thus it is, that moral forces are arrayed in deadly strife against the enemies of truth and honor all over the land. To yield is to lose all; and to contend, is often the signal for the betrayal of confidence and the desertion of friends. Error, borne along by the cohorts of the many, entraps the weak, enlists the reckless, and subsidizes the corrupt.

In the midst of causes so provocative of strife in the medical profession in this country, collisions must constantly occur. The degraded level to which medical education has been reduced, in many instances, by the multiplication of colleges, and the contentions and emulations between these for patronage by resorting to under-bidding and "complimentary tickets" for courses of instruction, with the selfish manipulations of the medical demagogue to over-reach his brethren in his efforts to secure practice, must ever be fruitful sources of professional troubles.

In Massachusetts, the profession has been convulsed. The State Medical Society had nurtured within its bosom, enemies to its peace and prosperity. Under its charter—in the name of "the Fathers" what business has a medical society with a charter?—anyman, be he Hindoo Jugler, or Hasheesh-Eater, had the legal right to membership provided he could pass the required examination. Through this gap numbers of Electics and Homœopathics gained admission. A few valient men, notwithstanding the opposition and resistance of the "Chancellors" of the society, brought the anomalous condition of the same before the American Medical Association with such results as compelled the society to take steps towards the expulsion of these irregulars. And here the second trouble commences. At this present time the charter is held by these parties as a shield over them, under cover of which they refuse to be expelled. So the matter stands. The good men of the profession see but two courses for their brethren of Massachusetts to persue; either to whip out these misguided irregulars, or to come out themselves and form a new society composed of the firm and consistent men of the profession in the State; and having done this to throw such barriers around the door of entrance and enact such laws as to forever preclude the admission of unprofessional innovators who may be unwilling to abide the ethics, however high their social position.

The trouble at Washington city was due to a want of respect, on the part of Dr. Bliss to the ethics, in that this Cundurango speculator refused to obey the admonitions of his society in his professional associations. This blissful gentleman of "pocket ethics," seduced by an unprofessional, consented to "affiliate" with him, for which act unrepented of, he was expelled. Under such a state of things Bliss "did not appreciate such dictation" and had he gotten up a society of his own, and then, with his followers "packed" his Association, he might have, in spite of Cundurango and the lesser ills of his other professional wanderings, declared that "peace" had been established "completely and fully" in Washington city. In addition to this, he might have folded his arms in complacency over his bosom, under the miserable fallacy that he had "aroused a new spirit in the medical profession" "that the deadly Upas of strife (poor Cundurango!) had been plucked up by the roots" (like his cancers) and that "the dawn of a better day (for disorganizers and quacks) was beginning to be clearly seen." But our brothers of Washington city were spared this infliction, though "beaten with" the "fewer stripes" of Cundurango.

In New York the Sayre-Ruppaner case has been the theme of professional and non-professional writers. The secular press of Gotham has teemed with versions of the case of Ruppaner vs. Sayre, and the Medical Society of the county of New York. And why? It is sometimes thought to be very important, when a Doctor is wrong in principle, to deceive the general reader at home, through the public press, in order to sustain his local position and interests. As to the merits of the Sayre-Ruppaner case —its origin—it is of small moment to inquire. The difficulty may or may not have been personal in its inception. The professional aspect of the case has, however, over-leaped every other. It has assumed a graver character. These gentlemen were members of the same society, Charges were preferred against Dr. R. by Dr. S. The case was brought before the *Comitia Minora,* for investigation. The Comitia, composed of honorable gentlemen, were, after eight months' deliberation, about to make its report, when Dr. Ruppaner had a writ of injunction issued against the Comitia, restraining its action, and thus carrying the matter before the courts!

Did Dr. Ruppaner do right, professionally, in thus setting his Society at defiance? Questions of a purely ethical character can never, from their very nature, become the subjects of adjudication by the courts of the country. Medical Societies are supposed to be composed of gentlemen of honor—are voluntary Associations, controlled and regulated by a higher, than human, law—a code of morals written upon the hearts and consciences of men, called the "Golden Rule." Upon this foundation rests the beautiful structure of medical ethics.

The high value of medical morals is recognized on every hand; for every offender against them, is loud in his proclamations of attachment and devotion to the ethics. Satan, to delude the good and seduce the innocent (if not to get into respectable company), has been known to clothe himself in garments of light.

But to return: Dr. Ruppaner, in our judgement is totally inexcusable in thus bringing reproach upon his Society, and in lifting the strong arm of the law, in a question of ethics, over the heads of his brethren. The comitia may have proceeded irregularly; may have meditated injustice toward him (which we cannot believe) and yet, were this all true, Dr. Ruppaner must fail to acquit himself before the profession of having taken the ethical (or honorable) course in order to throttle the report of the comitia. By so doing he seems to prejudge his case, and to enter up a verdict averse to his acquital. He should have awaited its decision and if against him, to have appealed to his Society (if possible) or have carried the case to his State Association, or failing there, to the Supreme Court of the Profession—the American Medical Association.

Notwithstanding the injunction, the Society, solely upon the unprofessional act in thus arresting the report of the comitia expelled Dr. Ruppaner—justly we think. On this occasion one of his fellow-members thus pointedly stated the merits of the case:

"Dr. Roosa said the question was simply this: Shall we allow a member accused of an offence—the accusation being properly made to the *Comitia Minora*—to scout its authority to investigate the case and report upon it to the Society, and to deny competence of the Society to decide upon it? Shall we allow him to go before the courts, and procure an injunction upon our regular mode of procedure in matters purely professional, and having no legal color whatever? It is exactly as if a church-member, accused of heretical doctrines or unchristian practices, should deny the church's authority to question him, and appeal to the law to restrain its investigations, and vindicate his orthodoxy or his godliness. If such a flagrant violation of its rules were to be allowed to pass without censure, all discipline in the Society was at an end, and it was time for it to dissolve."

Now, gentlemen of the Medical Society of New York, how will you treat Dr. Ruppaner, professionally? Will you suffer your members to recognize him in consultation and otherwise? Of course! The quarrel was "personal" and "should never have been carried" before your Comitia, and some of your would-be-self-constituted regulators of the profession in your city, may in defiance of the ethics, if "threatened with (such) professional degradation" (see transactions Georgia Medical Association, page 15,) by your "so-called" Society, when your society shall "refuse professional recognition," to Dr. R. and his affiliators, set up an independent Society, "not appreciating" your "authority, or in any way admitting the justice or propriety of such dictation" and determine "to continue the same professional intercourse which had previously existed between" them.

But gentlemen, you could not do this and be ethical, even "if threatened with" just "degradation." No, you could not. The copy-right to that "piece of acting" has been secured to parties in Georgia, and you could not infringe upon a claim so fully "established and enforced" down here. We cannot be charged with personal feeling in the "Sayre-Ruppaner" difficulty. We care not for men or parties—as such. We make no fight against individuals beyond the legitimate arena of the recognized code of ethics of America. To this code we desire, at all times, to render the strictest construction, and to bring to its impartial rule the greatest or humblest of the profession. If it censures we are bound to condemn; if it acquits we are delighted to approve.

The great lesson to be learned by the profession, is the one we have so often repeated. That the medical profession is a great brotherhood. That what affects its honor, integrity and interests in one locality, affects it in every other. The deplorable condition by which it is now surrounded, demands such action, as a tribute to its honor and glory. To be silent, because not locally interested, is to take sides with the wrong and to give impetus to the march of corruption and error.

